# Validation Study of Analytical Methods for Multiparameter Flow Cytometry-Based Measurable Residual Disease Assessment in Acute Myeloid Leukemia

**DOI:** 10.3390/ijms26104506

**Published:** 2025-05-08

**Authors:** Martina Barone, Agnese Patuelli, Michele Dicataldo, Maria Irno Consalvo, Gabriella Chirumbolo, Lorenza Bandini, Giulia Atzeni, Dorian Forte, Gianluca Cristiano, Emanuela Ottaviani, Antonio Curti, Francesco Buccisano, Lucia Catani, Mario Arpinati

**Affiliations:** 1 IRCCS Azienda Ospedaliero-Universitaria di Bologna, Istituto di Ematologia “Seràgnoli”, 40138 Bologna, Italy; agnese.patuelli@aosp.bo.it (A.P.); giulia.atzeni4@unibo.it (G.A.); emanuela.ottaviani@aosp.bo.it (E.O.); antonio.curti2@unibo.it (A.C.); lucia.catani@unibo.it (L.C.); mario.arpinati@unibo.it (M.A.); 2 Department of Medical and Surgical Sciences, University of Bologna, 40138 Bologna, Italy; michele.dicataldo@studio.unibo.it (M.D.); gabriell.chirumbolo2@unibo.it (G.C.); lorenza.bandini4@unibo.it (L.B.); dorian.forte2@unibo.it (D.F.); gianluca.cristiano3@unibo.it (G.C.); 3 Department of Biomedicine and Prevention, University of Rome Tor Vergata, 00133 Rome, Italy; irno.consalvo@gmail.com (M.I.C.); francesco.buccisano@uniroma2.it (F.B.)

**Keywords:** acute myeloid leukemia, measurable residual disease, multiparameter flow cytometry, leukemia-associated immunophenotypes

## Abstract

The standardization of multiparameter flow cytometry-based measurable residual disease (MFC-MRD) assessment in acute myeloid leukemia (AML) lacks clear criteria to define leukemia-associated immunophenotypes (LAIPs). In addition, the most specific/sensitive aberrations used to define LAIPs are often partially expressed by the leukemic clone at diagnosis, raising questions about their reliability for accurate MRD quantification. To address this, we investigated whether the quantification of LAIP+ cells reflects residual disease in cases of partial LAIP expression. The following two MFC-MRD approaches were evaluated by comparing their results to RT-qPCR for *NPM1* mutations: (1) the LAIP-method, wherein all cells within the patient-specific template created at diagnosis are counted without further gating; (2) the LAIP-based different-from-normal (DfN)-method, wherein cells+ for LAIP-specific aberrant markers are further selected. A total of 125 bone marrow samples from 25 *NPM1*-mutated AML patients were studied. Our data demonstrate that the LAIP-based DfN-method improves the MFC-MRD accuracy and comparability with molecular MRD. ROC analysis identified cut-offs of 0.034% and 0.095% to discriminate positive/negative results in patients receiving intensive chemotherapy and hypomethylating agents, respectively. We also found distinct accuracy degrees based on the LAIP-specific aberrant markers used for MRD assessment. These results refine the MFC-MRD method and highlight the importance of therapy-specific MRD cut-offs and LAIP classification based on specificity and sensitivity.

## 1. Introduction

Measurable residual disease (MRD) is defined as the persistence of leukemic cells after treatment that are undetectable by standard morphological examination. Its detection requires highly sensitive techniques and provides crucial information for assessing response to treatment and predicting relapse [1,2]. MRD is now well-established as an independent prognostic biomarker in patients with acute myeloid leukemia (AML) [3,4]. Several studies have demonstrated the impact of MRD on clinical outcome, showing that failure to achieve MRD negativity after induction therapy, or increased MRD levels during follow-up, predict morphological disease relapse [5,6,7].

MRD can be detected using both multiparameter flow cytometry (MFC) and molecular techniques such as reverse transcription-quantitative PCR (RT-qPCR) or next-generation sequencing (NGS). MFC is an applicable tool for evaluating MRD in up to 90% of AML patients [2,3,4,8], while only 40–60% of patients show a traceable molecular marker by RT-qPCR assays [9]. RT-qPCR reaches a sensitivity of 1 in 10^−4^–10^−6^ and can be employed in the presence of stable molecular alterations at diagnosis, such as *NPM1*-mutations or *RUNX1*-*RUNX1T1*, *CBFB-MYH11*, and *PML-RARA* fusion genes. Particularly, *NPM1*-based MRD has been shown to be a powerful independent prognostic factor of AML relapse, as it is a stable marker of AML status in most patients, and molecular relapse reliably predicts disease progression [1,3,4,10].

In contrast to molecular MRD, the detection of MFC-MRD in AML presents several challenges, mainly due to the phenotypic heterogeneity of the disease, which requires the determination of the leukemia-associated immunophenotype (LAIP) at diagnosis. AML cells have a highly variable phenotype, depending on the biology of the disease and the degree of differentiation. A standard CD34+/CD117+/CD13+/CD33+ AML phenotype does not lend itself to the monitoring of MRD, since this is also the phenotype of normal bone marrow (BM) hematopoietic stem cells (HSCs). The LAIP in AML is a patient-specific phenotype that can be characterized based on one or more of the following features: (1) asynchronous antigenic expression of immaturity/maturity biomarkers, such as CD34/CD117 with CD15 or CD11b; (2) aberrant lineage antigen expression, such as the lymphoid antigens CD19, CD7, CD4, CD25, CD2, and CD56; (3) and overexpression, reduced expression, or loss of antigens (CD123, CD33, CD13, HLA-DR) [11,12]. The quality of the LAIP for MRD tracking depends on the following: (1) specificity of the antigen combination and reduced presence of the identified LAIP in normal or regenerating BM cells [11]; (2) sensitivity in terms of the proportion of AML cells showing LAIP; (3) and stability of the LAIP based on immunophenotype changes during the course of the disease [9,11,13,14,15]. In the presence of a robust LAIP, it is possible to detect one LAIP+ cell in 10,000 [16].

The European LeukemiaNet (ELN) recommendations define the entire process of MRD assessment, from sample collection to result reporting, including the standard markers to be used [1,2,3,4,12]. Regarding MFC data analysis/interpretation, the ELN guidelines recommend using a combination of the LAIP and the different-from-normal (DfN) approach to exploit the advantages of both methods. The LAIP approach identifies the aberrant phenotype at diagnosis and tracks it during follow-up, while the DfN approach identifies aberrant antigen expression patterns not expressed by normal HSCs during follow-up. The LAIP approach is specific but does not consider immunophenotypic shifts, and cannot be used when no LAIP is identified at diagnosis. The DfN approach can detect emerging LAIPs, but the aberrations observed may not be disease-specific, and may be present in minute amounts in healthy or regenerating BM, leading to false-positive results.

Additionally, the criteria required to define a LAIP have not yet been clarified. In particular, specific combinations of LAIPs, as well as the role of the number and typology of LAIPs on the sensitivity of MRD, have not been defined. Moreover, the thresholds used to assess the MFC-MRD range from 0.035 to 0.2% without considering the large number of LAIPs, their differences in specificity/sensitivity, and the clinical context of MRD time points.

Several studies have evaluated the specificity of different LAIPs, identifying LAIPs that are more specific than others for MRD monitoring, but reporting conflicting data [8,11,13,14,15,17,18,19]. Despite this, aberrant lineage antigen expression remains the most robust and reliable feature for distinguishing residual AML cells from normal HSCs [12]. In principle, the LAIP-method measures the most prominent LAIP+ cell population at diagnosis, which is then monitored during therapy [12]. However, there is no consensus on the minimum percentage of LAIP expression that the leukemic clone should exhibit, at diagnosis, to select biomarkers for MRD detection. Moreover, in most AMLs, the more specific LAIPs (expression of aberrant lineage markers) are often only partially expressed by leukemic cells at diagnosis. Therefore, in the MRD analysis, selecting only cells positive for aberrant antigens could not include part of the residual leukemic clone, which is apparently free from phenotypic alterations.

In this study, we aimed to evaluate whether the partial expression of aberrant lineage markers (LAIPs) by the leukemic clone at diagnosis can be reliably used for MRD detection in *NPM1*-mutated AML. To address this issue, we applied a patient-specific gating strategy to track residual leukemic cells, and we compare the following two MFC-MRD analytical approaches: (1) the LAIP-method, which identifies all cells within the patient-specific template as MRD cells, and (2) the LAIP-based DfN-method, which specifically selects cells expressing LAIP-specific aberrant lineage markers. To assess the accuracy of both approaches, we compared the MFC-MRD results to the RT-qPCR-based MRD quantification of the *NPM1* mutation, a validated and highly sensitive molecular marker for disease monitoring in AML [2,6,20,21,22,23].

## 2. Results

### 2.1. Study Design and MRD Analysis Overview

**Figure 1:**
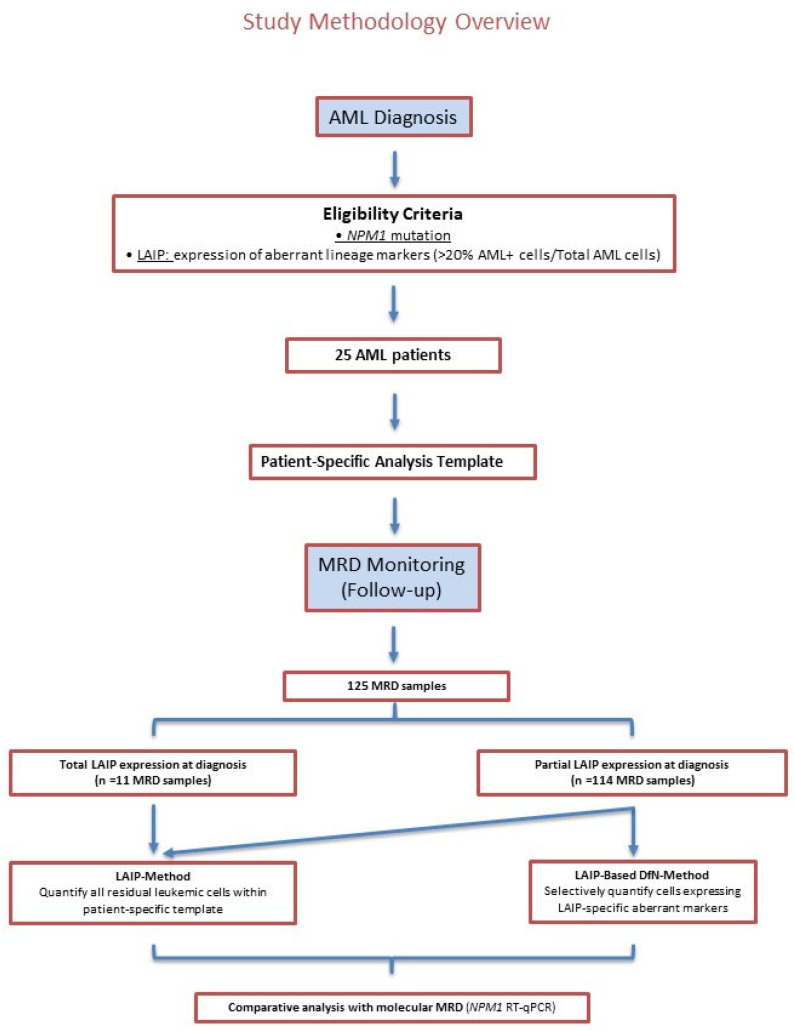
Flow chart illustrating the selection of AML patients, the application of two MFC-MRD approaches (LAIP- and LAIP-based DfN-methods) for MRD monitoring, and the comparison to RT-qPCR quantification of *NPM1*-mutated transcripts.

In this study, we specifically focused on the challenge posed by the partial expression of LAIPs at diagnosis and its impact on MRD monitoring. Indeed, partial LAIP expression may compromise the accuracy of MRD detection if it is not adequately addressed. To explore this issue, we designed a study aimed at evaluating whether partial LAIP expression can still be reliably used for MRD assessment in *NPM1*-mutated AML. We provide here a concise overview of the study design and the analytical strategies adopted for MRD assessment by MFC. A detailed description is provided in the Section 4. A flow chart illustrating the selection of AML patients and the analysis process of MRD samples is shown in Figure 1.

A total of 25 patients with newly diagnosed *NPM1*-mutated AML were prospectively enrolled. Inclusion criteria were as follows: (i) availability of a diagnostic BM sample; (ii) confirmed *NPM1* mutation; and (iii) the presence of a clearly identifiable LAIP suitable for MFC-MRD monitoring. Patients were followed throughout treatment and clinical follow-up. Clinical features of AML patients are summarized in Table S1. Patient-specific immunophenotype and LAIP aberrations selected for MRD assessment are reported and highlighted in Table 1.

**Figure 2:**
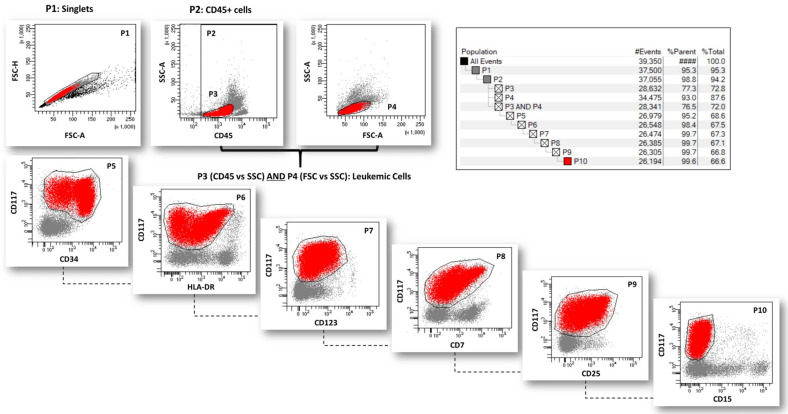
Gating strategy to create a specific analysis template for an AML patient at diagnosis. Representative example of the gating strategy used to select/identify and characterize the leukemic population (Pt#8, Table 1; Panel 3, Table S2), including doublets exclusion (P1), definition of white blood cells (WBCs) as CD45^+^ cells (P2), selection of leukemic blasts based on CD45 expression (P3) and FSC vs. SSC features (P4), and clustering of immunophenotypic characteristic of AML cells by sequential gates (from P5 to P10). Sequential gates must include the total leukemic population and absence or partial and total expression of markers.

At diagnosis, leukemic cells were characterized by MFC, and a patient-specific analysis template was generated using a sequential and hierarchical gating strategy to cluster all immunophenotypic features (total, partial, and absent expression of all markers) of the leukemic clone. An example of an analysis template is shown in Figure 2.

**Figure 3:**
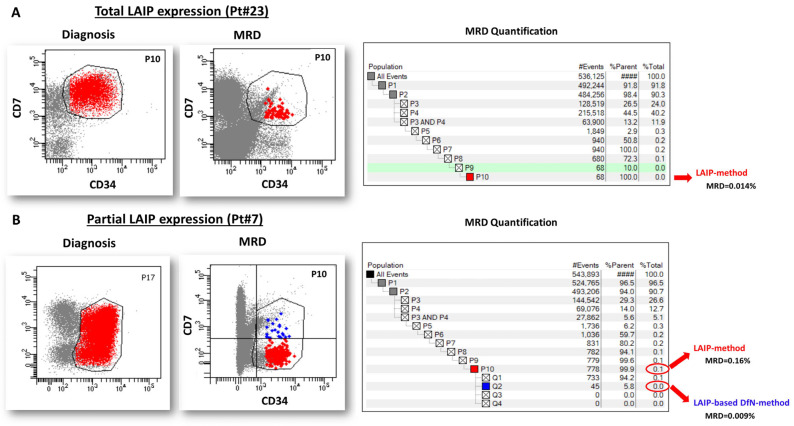
Description of the two MFC-MRD analytical methods. (**A**): MRD quantification in the cases of total LAIP expression of AML cells at diagnosis (90–100% of AML cells, Pt#23, Table 1). After applying the patient-specific analysis template, the cell population included in the last gate of the hierarchy (in this case, P10) is directly quantified and identified as MRD cells (LAIP-method). (**B**): MRD quantification in the cases of partial LAIP expression of AML cells at diagnosis (20–90%, Pt#7, Table 1). Using the patient-specific analysis template, the cell population included in the last gate of the hierarchy (in this case, P10) is analyzed. The LAIP-method quantifies all cells included in gate P10 as MRD cells, while the LAIP-based DfN-method selects only cells positive for the LAIP-specific aberrant marker expressed at baseline (LAIP+ cells).

The patient-specific analysis template was applied to follow-up BM samples for MRD monitoring using the following two approaches: (1) the LAIP-method, quantifying all residual leukemic cells within the patient-specific analysis template without further gate manipulation, and including both positive and negative cells for aberrant lineage markers; (2) the LAIP-based DfN-method, selectively quantifying cells expressing LAIP-specific aberrant lineage markers. If the LAIP was totally expressed by AML cells at diagnosis, MRD monitoring was performed using only the LAIP-method, whereas if the LAIP expression was partial, both the LAIP and the LAIP-based DfN-methods were applied and compared. Examples of the two analytical methods are shown in Figure 3.

A total of 125 MRD samples were analyzed. Samples were collected at variable timepoints according to treatment protocols and clinical management decisions. All available MRD samples were included, as the aim was to evaluate the analytical performance of the two MFC-MRD approaches independently of the sample collection timing. MFC results were compared to the RT-qPCR quantification of *NPM1*-mutated transcripts. The total number of MRD samples per patient, and the treatments received, are detailed in Table 1.

### 2.2. Comparison of the Concordance of the Two MFC Analytical Methods Results with NPM1-MRD Outcome

**Figure 4:**
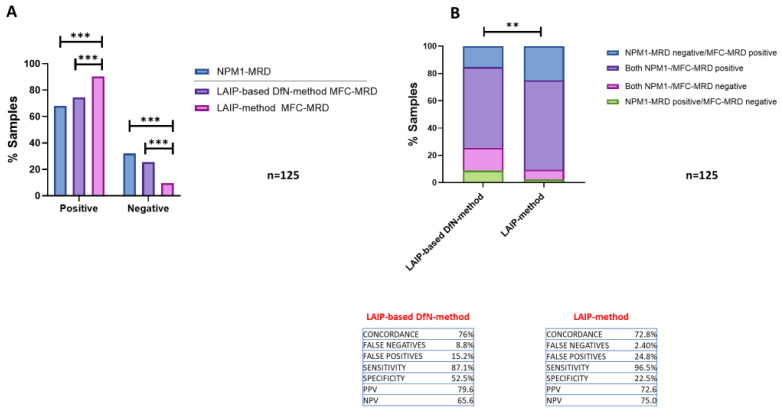
Comparison of MRD results obtained by RT-PCR for *NPM1* mutations (*NPM1*-MRD) and the two MFC-MRD approaches. (**A**): Representative histogram of the percentage of positive/negative MRD samples (*n* = 125) obtained with *NPM1*-MRD and MFC-MRD, using LAIP- and LAIP-based DfN-methods (Chi-square test; *** *p* ≤ 0.001). (**B**): Concordance between the LAIP- and LAIP-based DfN-method results and the *NPM1*-MRD outcomes. The results are shown as stacked histogram and expressed as percentage of both positive/negative (purple/pink) and false-positive/negative (blue/green) MRD samples (Chi-square test; ** *p* ≤ 0.01). The percentages of concordance, false-positive/negative, sensitivity and specificity, and of the positive/negative predictive value (PPV, PNV) of the MFC analytical methods are reported.

Of the 125 MRD samples studied, the molecular MRD analysis revealed that 85 samples (68%) were *NPM1*-positive (i.e., >0.01 *NPM1*-mutated ratio), whereas 40 samples (32%) were negative. MRD analysis was performed using both of the MFC methods, as follows: 113 samples (90.4%) were detected as MRD-positive using the LAIP-method, whereas 93 (74.4%) tested MRD-positive using the LAIP-based DfN-method. The percentage of positive MRDs determined by the LAIP-method was significantly increased compared to that detected by LAIP-based DfN-method (*p* = 0.0009) or molecular *NPM1*-MRD (*p* < 0.0001) (Figure 4A).

A final concordance rate of 76% (*n* = 95) was calculated between the LAIP-based DfN-method and the *NPM1*-based results as compared to 72.8% between the LAIP-method and the *NPM1*-based results (*n* = 91) (Figure 4B) (Chi-square test; *p* = 0.0054). Comparing the two MFC methods, the LAIP-method showed an increased false-positive rate of 24.8% (*n* = 31) as compared to 15.2% (*n* = 19) with the LAIP-based DfN-method; conversely, the LAIP-based DfN-method revealed an increased false-negative rate of 8.8% (*n* = 11) as compared to 2.4% (*n* = 3) with the LAIP-method. It is of note that three samples resulted as falsely negative with both MFC methods, probably due to the reduced sensitivity of the MFC technique compared to RT-qPCR.

**Figure 5:**
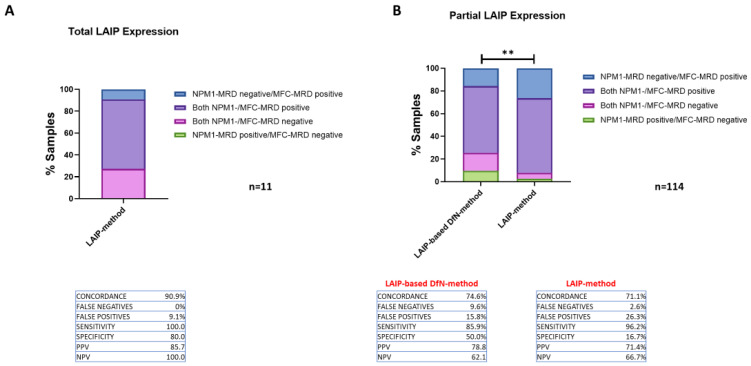
Comparison of MRD results obtained by RT-PCR for *NPM1* mutations (*NPM1*-MRD) and the two MFC-MRD approaches according to the percentage of LAIP expression (total or partial) of AML cells at diagnosis. (**A**): Concordance between the results of MFC-MRD (LAIP-method only) and *NPM1*-MRD in the evaluation of MRD samples from patients with total expression (90–100% of AML cells) of LAIP-specific aberrant lineage markers in AML cells at baseline (*n* = 4 patients; *n* = 11 MRD samples). (**B**): Concordance between LAIP- and LAIP-based DfN-methods and NPM1-MRD results in the evaluation of MRD samples from patients with partial expression (20–90% of AML cells) of LAIP-specific aberrant lineage markers in AML cells at baseline (*n* = 21 patients; *n* = 114 MRD samples). The results are shown as a stacked histogram and expressed as a percentage of both positive/negative (purple/pink) and false-positive/negative (blue/green) MRD samples (Chi-square test; ** *p* ≤ 0.01). The percentages of concordance, false-positive/negative, sensitivity and specificity, and of the positive/negative predictive value (PPV, PNV) of the MFC analytical methods are reported.

Eleven MRD samples studied were from four patients with a LAIP characterized by the complete expression of aberrant lineage markers (CD7 or CD25) on leukemic cells at diagnosis. In these cases, the two methods are expected to provide equivalent results, as the analysis template already includes all cells positive for the aberrant marker, without the need to restrict the analysis (as shown in Figure 3). It should be noted that the MFC- and *NPM1*-MRD results of these samples showed a concordance of 90.9% (*n* = 10), with 9.1% (*n* = 1) being false-positives, and without false-negatives (Figure 5A). Conversely, a lower concordance rate was observed in MRD samples from AML patients (*n* = 114) with a phenotypic profile, showing only partial expression of one or more aberrant markers on leukemic cells at diagnosis (Figure 5B). In this scenario, the LAIP-based DfN-method demonstrated a 74.6% (*n* = 85) concordance rate, which was higher than the LAIP-method, at 71.1% (*n* = 81) (Chi-square test; *p* = 0.0029). Once again, the LAIP-based DfN-method revealed a higher percentage of false-negatives than the LAIP-method (9.6% (*n* = 11) vs. 2.6% (*n* = 3), respectively), while the LAIP-method showed an increased proportion of false-positives (26.3% (*n* = 30) vs. 15.8% (*n* = 18), respectively).

Taken together, these data showed a significant difference between the results of the two MFC methods, mainly in the proportion of false-positive/negative results. The LAIP-based DfN-method appeared to have a slightly higher concordance with *NPM1*-MRD results and higher specificity than the LAIP-method, while the LAIP-method showed higher sensitivity but lower specificity.

### 2.3. Comparing the Accuracy of Two MFC Approaches for MRD Quantification

To evaluate the accuracy of the two MFC methods and determine which is more effective for MRD assessment in AML, we performed a ROC analysis.

Although MRD tests are typically quantitative, results are often reported as positive or negative. This quantitative measurement can be converted into a binary (1 = positive/0 = negative) classification. Therefore, we used *NPM1*-MRD results as a reference to categorize MRD samples accordingly.

Since the accuracy of an MRD method depends on its ability to distinguish between positive and negative cases, we examined how the blast percentages estimated by the two MFC methods were distributed according to the *NPM1*-MRD classification.

**Figure 6:**
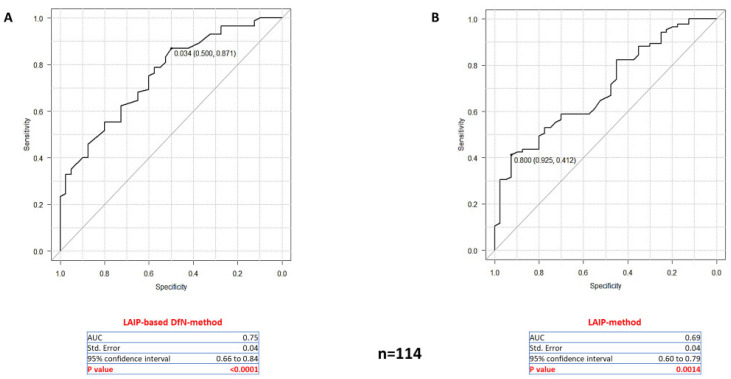
Accuracy of the two MFC-MRD analytical methods in evaluating MRD samples from patients with partial expression (20–90% of AML cells) of LAIP-specific aberrant markers in AML cells at diagnosis (*n* = 21 patients; *n* = 114 MRD samples). (**A**,**B**): ROC curves obtained by distributing the leukemic blast percentages estimated from the LAIP-based DfN- and LAIP-methods according to the *NPM1*-MRD outcome. Area under curve (AUC), Standard Error, 95% confidence interval, and *p*-value of each ROC curve were reported.

Firstly, we analyzed MRD samples from patients with partial expression of aberrant markers on leukemic cells at diagnosis (*n* = 114) and evaluated how the two methods performed in differentiating these cases (Figure 6A,B). The ROC curves obtained showed that the LAIP-based DfN-method had higher accuracy, as evidenced by a higher area under the curve (AUC = 0.75; *p* < 0.0001), compared to the LAIP-method (AUC = 0.69; *p* = 0.0014). Additionally, we observed that the optimal cut-off for MRD positivity assessed by the LAIP-based DfN-method was 0.034% of LAIP-positive cells (specificity = 50%; sensitivity = 87.1%), while the LAIP-method had a cut-off of 0.8% (specificity = 92.5%; sensitivity = 41.2%).

Next, we focused on samples with a total blast percentage (CD34+/CD117+) of less than 1% (*n* = 93) in MFC analysis and a NPM1 ratio of less than 1 (*n* = 84), which can be considered the true MRD assessments. Both evaluations confirmed that the LAIP-based DfN-method had a higher concordance rate with *NPM1* results (Figure S1A,B; Chi-square test: *p* = 0.0063 and *p* = 0.009; respectively) and AUC (Figure S1C,D) than the LAIP-method. Notably, both ROC curves identified a cut-off of 0.034 for the LAIP-based DfN-method (specificity = 54.1%; sensitivity = 82.1%, and specificity = 51.3%; sensitivity = 84.4%; respectively).

Overall, the ROC analysis confirmed that the LAIP-based DfN-method is statistically more accurate in identifying positive/negative patients with a cut-off that is consistent with a previous work [24].

### 2.4. Accuracy of MFC-MRD Assessment According to Therapy

**Figure 7:**
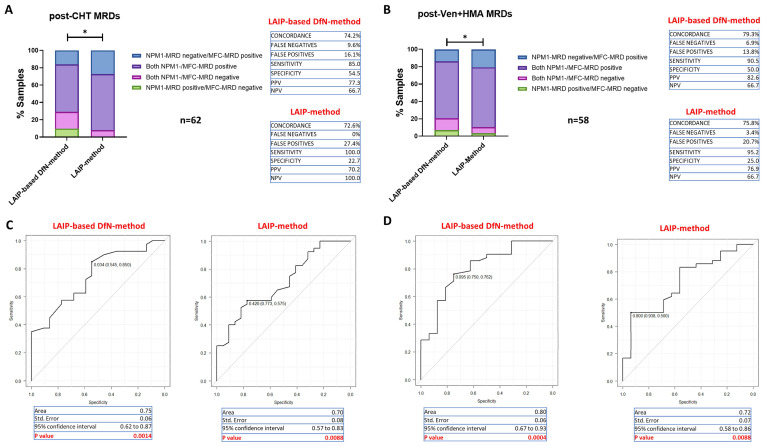
Accuracy of the two MFC-MRD analytical methods according to therapeutic setting. (**A**,**B**): Concordance between the results of MFC-MRD, using LAIP- and LAIP-based DfN-methods, and *NPM1*-MRD in evaluating MRD samples from patients receiving intensive chemotherapy regimens (post-CHT MRDs, *n* = 62) and Ven+HMA-based therapies (post-Ven+HMA MRDs, *n* = 58). The results are shown as a stacked histogram and expressed as a percentage of both positive/negative (purple/pink) and false-positive/negative (blue/green) MRD samples (Chi-square test; * *p* ≤ 0.05). The percentages of concordance, false-positive/negative, sensitivity and specificity, and of the positive/negative predictive value (PPV, PNV) of the MFC methods are reported. (**C**,**D**): ROC curves obtained by distributing the leukemic blast percentages estimated from the LAIP- and LAIP-based DfN-methods according to the *NPM1*-MRD outcome, analyzing post-chemotherapy (post-CHT MRDs, *n* = 62) and post-Ven+HMA-based therapy (post-Ven+HMA MRDs, *n* = 58) MRD samples. Area under curve (AUC), Standard Error, 95% confidence interval, and *p*-value of each ROC curve were reported.

Next, we evaluated whether MFC-MRD analysis might be affected by treatment type. Thus, we evaluated the reliability of the two MFC-MRD approaches in patients receiving intensive chemotherapy (CHT) regimens (*n* = 62 MRD samples) or Ven+HMA-based therapies (*n* = 58 MRD samples). As shown in Figure 7, the LAIP-based DfN-method confirmed a higher concordance rate (Figure 7A,B; Chi-square test: *p* = 0.0108 and *p* = 0.02; respectively) and accuracy (AUC) (Figure 7C,D) compared to the LAIP-method in both clinical settings.

Similarly, using the LAIP-based DfN-method (Figure 7C,D), ROC analysis identified a cut-off of 0.034% for post-CHT MRD samples (AUC = 0.75; *p* = 0.0014) (specificity = 54.5%; sensitivity = 85%), whereas the cut-off for Ven+HMA-associated MRD samples was 0.095% (AUC = 0.80; *p* = 0.0004) (specificity = 75%; sensitivity = 76.2%). Conversely, the LAIP-method showed lower accuracy with higher cut-offs in both clinical conditions.

These results demonstrate that the LAIP-based DfN-method may be considered the most reliable in both clinical contexts. However, distinct cut-off values need to be set according to the therapeutic settings.

### 2.5. Evaluation of the Reliability of Distinct LAIP-Specific Aberrant Lineage Markers in MFC-MRD Monitoring

As reported in Table 1, in our cohort (*n* = 25), 21 patients showed LAIPs characterized by the partial expression of the aberrant lineage markers (CD7, CD4, CD56, and CD25) on AML cells at diagnosis. Consequently, we investigated the reliability of the LAIP-specific aberrant markers used for each MFC-MRD assessment. We also compared the two MFC methods to identify the most appropriate approach (Figure S2A,B).

Regarding CD7 marker-based MRD assessments (*n* = 33), the LAIP-based DfN-method showed a lower concordance rate (72.7%) compared to the LAIP-method (81.8%). Notably, most discrepancies of the LAIP-based DfN-method were false-negatives (24.2%), potentially attributed to the loss of the antigen in residual leukemic cells.

Conversely, CD4 marker-based MRD assessments (*n* = 59) had a generally low concordance rate, albeit higher with the LAIP-based DfN-method (71.0%) than with the LAIP-method (64.5%), given the latter’s high false-positive rate (35.5%).

It is of note that MRD assessments based on both CD56 (*n* = 11) and CD25 (*n* = 11) markers showed the highest concordance with *NPM1*-MRD results, respectively. Specifically, CD56 marker-based MRD assessments showed a concordance rate of 90.9% with both MFC methods, while CD25 marker-based MRD assessments showed a concordance rate of 100% and 90.9% with the LAIP-based DfN- and LAIP-methods, respectively.

Finally, we performed ROC analysis for the most frequent aberrant lineage markers, namely CD7 and CD4 (Figure S3A,B). The LAIP-based DfN-method showed a higher accuracy (AUC = 0.81 *p* = 0.025) in CD7 marker-based MRD analyses than the LAIP-method (AUC = 0.68; *p* = 0.209), and ROC analysis identified a cut-off of 0.047% (specificity = 100%; sensitivity = 64.3%). Conversely, a lower accuracy was found for CD4 marker-based MRD analyses, using both the LAIP-based DfN- (AUC = 0.66; *p* = 0.032) and LAIP- (AUC = 0.62; *p* = 0.112) methods. However, the LAIP-based DfN-method appeared to be the best approach, using a cut-off of 0.035 (specificity = 43.5%; sensitivity = 84.6%).

Our data therefore revealed different degrees of accuracy based on the LAIPs used for MRD assessment.

## 3. Discussion

MRD monitoring plays a key role in the standard of care of AML patients. Nowadays, monitoring of specific genetic mutations represents the most sensitive method [10^−4^–10^−6^] to estimate MRD, but its applicability is limited [3,4,25,26,27]. The detection of LAIP+ cells by MFC has been an attractive alternative to molecular monitoring. Due to its broad applicability, interest in using the MFC as a tool to measure MRD is progressively increasing worldwide, and there is a concomitant demand for harmonization and standardization [4]. Lack of harmonization in technical approaches of MFC-MRD evaluation may render MRD data comparison between studies challenging, as shown in some meta-analyses [28], and it may also reduce its prognostic value [29]. To date, several studies have demonstrated the predictive significance of MFC-MRD in patients with AML, without considering that the MFC-MRD technique still needs validation [13,18,19,30,31,32,33]. In contrast, Rossi et al. showed a low diagnostic performance in the MFC method and a high rate of relapse in MRD-negative patients, mainly determined by low sensitivity [11,34]. Additionally, MFC data are often discordant with molecular MRD results, making their interpretation challenging in clinical practice. Although many studies tried to improve MRD assessment, the accuracy of MFC-MRD in reflecting disease status and identifying relapse is still unsatisfactory.

Recent studies aimed to improve this methodology, focusing on the standardization of antibody panels and the validation of an MFC-MRD method, possibly including automated methods for the analysis and interpretation of MFC-MRD data [4,35,36,37]. Tettero et al. validated a semiquantitative MFC-MRD assay [38]. For validation, their method was compared to an alternative flow cytometry test routinely used to detect hematologic malignancies. The results showed a high accuracy of the MFC-MRD test in correctly quantifying LAIP at diagnosis and MRD at follow-up. However, the main limitation was the lack of an adequate reference test [4].

Wang et al. also validated an MFC-MRD method by comparing the MRD test data to concomitant molecular genetic analysis [39]. However, these studies did not use the LAIP approach; therefore, contrary to our method, the MRD assays do not refer to an analysis template developed at diagnosis, which makes the test less patient-specific. Consequently, these studies do not address the challenge of LAIPs that are partially expressed at diagnosis.

Based on the *NPM1*-MRD results of AML patients, here we examined two different MFC-MRD analysis approaches to evaluate whether the selection/quantification of cells expressing aberrant lineage markers reflected residual disease. Four patients showed LAIPs representing 90–100% of AML cells at diagnosis, and their MRD results were highly accurate, underscoring the significant role of the percentage of LAIP+ cells at diagnosis. In patients with partial LAIP expression in AML cells at baseline (20–90% of AML cells), the accuracy of the MRD assay decreased. Despite that, we demonstrated that restricting the MRD analysis to the cells positive for the LAIP-specific aberrant lineage markers (LAIP-based DfN-method) could improve the performance of MFC-MRD in terms of concordance with *NPM1*-MRD. Conversely, when we enumerated the total cells included in the patient-specific template (LAIP-method), even though it contains all the characteristics of the leukemic clone, we potentially included healthy and regenerating cells, resulting in false-positive results. In contrast, the LAIP-method has the potential to simplify or better standardize MFC-MRD, as it requires a less elaborate and less operator-dependent analysis. Hanekamp et al. also postulated an alternative MRD approach that allowed MRD to be quantified more objectively [40]. It is known that CD34- and CD117-positive leukemia compartments contain normal progenitors and leukemia-initiating and propagating cells. Therefore, they hypothesized that the quantification of total CD34+/CD117+ cells (AML + normal HSCs) might be equally informative for relapse initiation as the total leukemic load. Consistent with our results, they showed that the risk of relapse does not correlate with the total blast rate, but rather with the leukemic part of the total progenitor population.

Venditti et al. [30,31] showed that the persistence of LAIP-positive cells >0.035% after consolidation, but not after induction, is significantly correlated with a high probability of subsequent relapse and shortened overall and relapse-free survival. Consistent with their findings, we identified a similar cut-off value of 0.034%, using the LAIP-based DfN-method. This value yielded the highest concordance rate between MFC-MRD and *NPM1*-MRD results, identifying MRD positive/negative patients with 75% accuracy (AUC = 0.75). Consequently, based on the results of Venditti et al., we anticipate that this analytical approach may also predict relapse.

Recently, the implementation of treatment protocols with non-intensive regimens based on hypomethylating agents (HMAs) set up new issues in monitoring MRD. In contrast to post-chemotherapy MRD assessments, the detection of MFC-MRD after HMAs and its prognostic value are not well-defined [41]. Effectively, our data confirm a cut-off of 0.034% for MRD assays performed after chemotherapy and suggest that a cut-off of 0.095% should be considered after Ven+HMA-based therapies. HMAs have different mechanisms of action and dynamics of response as compared to intensive conventional chemotherapy. Specifically, HMAs restore the normal expression of genes that play a critical role in cell differentiation, altering the immunophenotype of the leukemic clone and making the evaluation of MFC-MRD more difficult. Indeed, this could be the reason why our analysis identified a higher cut-off compared to that defined for post-chemotherapy MRD assays.

As also suggested by Rossi et al. [11], these data underscore the need to define a classification of LAIPs based on their specificity and sensitivity. LAIPs harboring markers such as CD25 and CD56 may allow for very accurate MFC-MRD analyses with high specificity and sensitivity. Conversely, aberrant lineage markers such as CD7 and CD4 may be less reliable. The CD4 marker, albeit expressed by a high percentage of leukemic cells at diagnosis (55–85% of AML cells, see Table 1), does not appear to specifically reflect residual disease, resulting in many false-positive results. In contrast, the CD7 marker makes the LAIP specific but less sensitive, resulting in a high false-negative rate.

Therefore, our results highlight the importance of the correct choice of LAIPs for MRD analysis, and show that the LAIP-based DfN-method remains the most appropriate analytical approach.

## 4. Materials and Methods

### 4.1. Patients and Samples

BM samples obtained from 25 *NPM1*-mutated AML patients at diagnosis were characterized to identify LAIP. A total of 125 BM samples obtained from these patients during follow up were studied for MRD by both MFC and RT-qPCR of *NPM1* mutations. Sixty-two MRD evaluations were performed on BM from patients receiving chemotherapy (CHT) regimens; fifty-eight on BM from patients receiving Venetoclax (Ven) in combination with hypomethylating agents (HMAs), such as azacitidine (AZA) or decitabine (DAC); and five after allogeneic stem cell transplant (allo-SCT). The MRD samples were collected at different timepoints depending on the treatment schedules and patient-specific clinical management. All available MRD samples were included, regardless of the specific timepoint, since the aim of this study was to evaluate the accuracy of the MFC-MRD methods independently of the timing of sample collection. Clinical features of AML patients are summarized in Table S1. All samples were collected with written informed consent and after the local ethic board approved the study. The research was approved by the institutional review board of the Area Vasta Emilia Centro (AVEC) Ethical Committee (approval code: 94/2016/O/Tess).

### 4.2. Immunophenotyping of AML at Diagnosis

BM samples were collected in EDTA from patients with AML (n = 25) at diagnosis, and the leukemic cells were characterized by MFC. Consistent with published evidence [1,2,3,4,12,24], immunophenotyping of AMLs at diagnosis was performed using 4 panels of 8-color monoclonal antibodies (MoAb), which included the mandatory and additional markers specified by the European LeukemiaNet guidelines (for details see Table S2). Panel 1 was mainly used to determine the lineage of acute leukemia and, in the case of AML, to assess the expression of aberrant lineage markers such as CD2, CD10, CD19, and CD5 that are rarely expressed in AML. It is of note that none of these markers were expressed in our patient cohort. Panels 2 and 3, specifically used for MRD assessment, include a consistent set of four backbone markers, such as CD45, CD34, CD117, and HLA-DR. This design facilitates accurate identification and quantification of leukemic cells and ensures reliable overlap between panels [12]. Furthermore, this combination provides CD45 for white blood cells (WBCs) gating, primitive markers (CD34, CD117), and myeloid markers (HLA-DR as well as CD13, CD33 in Panel 2) to highlight the leukemia cell population. Additional MoAbs were used to detect aberrant expression of the most frequent lineage markers (CD4, CD7, CD25, CD56), asynchronous antigen expression (CD15 and CD11b), and antigen overexpression (CD123). To characterize the monocytic/myelomonocytic component, we added a monocytic tube, consisting of CD64, CD11b, CD14, CD4, CD34, HLA-DR, CD33, and CD45 (Panel 4, Table S2). However, no cases of monocytic AML were identified in our study. All MoAbs were used for surface staining and were purchased from BD Biosciences (Berkeley, CA, USA). Stained samples were acquired using BD FACSCanto^TM^ II (BD biosciences, Berkeley, CA, USA).

According to the guidelines [1,2,3,4,12], identification of leukemic cells was performed by discriminating doublets (FSC-A versus FSC-H), discarding debris, and checking viability on an FSC/SSC scatterplot. Subsequently, after defining WBCs as the CD45+ population, the leukemic population was selected by combining CD45 expression with primary markers (CD34, CD117) and myeloid markers (HLA-DR and CD13, CD33, Panel 2 and 3). For each patient at diagnosis, the percentage of leukemic cells on the WBCs was quantified (Table 1) and the values obtained from the analysis of Panels 2 and 3 were checked for overlapping.

We used the LAIP approach [2,12]; therefore, once the leukemic population was selected, we assessed phenotypic aberrations to identify LAIP. We evaluated the percentage of positive leukemic cells for each marker (AML+ cells/Total AML cells, Table 1), referring to the internal negative/positive controls (internal negative/positive controls are listed in Table S3) [38,42]. Several studies [8,11,13,14,15,17,18,19] have shown that the aberrations characterized by the combined expression of myeloid markers with lineage markers (for example, CD4, CD7, CD56, and CD25) are the most specific and sensitive alterations (LAIPs) that allow for distinguishing AML cells from healthy or regenerating HSCs in MRD evaluation. Therefore, in our study, LAIPs were defined based on the expression of aberrant lineage markers and considered relevant only if expressed by more than 20% of leukemic cells. Moreover, since antigen expression can shift during therapy, when possible, we tried to identify more than one aberrancy for each patient at diagnosis. Patient-specific immunophenotype and LAIP aberrations selected for MRD assessment are reported and highlighted in Table 1. Based on the LAIPs identified in our patient cohort, Panels 2 and 3 (Table S2) were used to cluster the leukemic clone features and for MRD assessment.

Since the MRD gating strategy requires hierarchical gating that includes all phenotypic features expressed by the leukemic clone at diagnosis [12], a patient-specific analysis template was created for both Panels 2 and 3 using the sequential gating technique. In principle, sequential gates include the entire leukemic clone and all phenotypic features (absence or partial and total expression of all markers). The templates of Panels 2 and 3 were checked to confirm overlapping percentages of AML cells. An example of an analysis template is shown in Figure 2. The patient-specific analysis template was created using the BD FACSDiva v 9.0 software (BD biosciences, Berkeley, CA, USA). The same combinations of MoAbs and fluorescence (Panels 2 and 3), and the patient-specific analysis template, were used to track and quantify residual LAIP+ cells in MRD evaluation.

### 4.3. Flow Cytometric Evaluation of MRD

BM samples (n = 125) were processed according to the EuroFlow standard operating protocol for MRD evaluation [2,12,24]. We used the stain/lyse/wash approach for preparing BM samples for MFC evaluation. Each MRD sample was stained using Panel 2 and 3 (Table S2) to create the analysis template at diagnosis. After staining, a minimum of 500,000 cells (excluding doublets, debris, and CD45-negative cells) were acquired using the BD FACSCanto^TM^ II flow cytometer (BD biosciences, Berkeley, CA, USA) and analyzed with BD FACSDiva software v 9.0 (BD biosciences, Berkeley, CA, USA). When it was not possible to acquire 500,000 events, we acquired as many events as possible. The patient-specific analysis template prepared and stored at baseline was applied for MRD analysis. Adjustments of gates may be necessary for each individual specimen. Once again, the templates of Panels 2 and 3 were checked to confirm overlapping percentages of cells.

Since the analysis template is already built at diagnosis to include the absent, partial, or total expression of aberrant lineage markers by leukemic cells, the template will include both LAIP-positive and negative cells if LAIP was partially expressed, as opposed to only LAIP-positive cells if LAIP was fully expressed at diagnosis. Therefore, in cases where LAIP was partially expressed at baseline (*n* = 21 patients; *n* = 114 MRD samples), we investigated two different approaches to quantify MRD cells, as follows: the LAIP-method, which considers all cells identified by the patient-specific analysis template without further gate manipulation; the LAIP-based DfN-method, which specifically filters out cells positive for LAIP-specific aberrant lineage markers identified and expressed at diagnosis. If multiple aberrant lineage markers were present, we prioritized those with the highest expression levels in AML cells at baseline. Conversely, in cases where LAIP was fully expressed at baseline (*n* = 4 patients; *n* = 11 MRD samples), we used the LAIP-method exclusively. Examples of the two analytical methods are shown in Figure 3**.**

Therefore, the LAIP-based DfN-method applies a filtering strategy inspired by the DfN approach, but instead of identifying general phenotypic abnormalities relative to normal HSCs, it specifically selects aberrant lineage markers associated with the LAIP profile established at diagnosis.

The threshold for discriminating positive/negative-MRD was set at 0.035% of residual leukemic cells (LAIP+ cells) calculated on the CD45+ leukocyte population (excluding CD45− cells) [16,30,31].

### 4.4. Patterns and Ranges in Normal and Regenerating Bone Marrow Samples

To define whether the expression of the antigens used for MRD analysis is aberrant, and to establish “in house” reference ranges, we evaluated the performance of each aberrant phenotype (LAIP) in normal and regenerating BM samples. Specifically, we analyzed the aberrant antigens most commonly expressed by AML cells in our patient cohort, as follows: aberrant lineage antigens (CD7, CD4, CD25, and CD56); asynchronous antigenic expression of biomarkers of immaturity/maturity, such as CD34/CD117 with CD15; and overexpression of antigens (CD123).

We analyzed the frequency of LAIPs in 7 normal BM samples from patients with lymphomas at diagnosis without BM involvement and in 9 regenerating BM samples from patients with Acute Lymphoblastic Leukemia (ALL) in remission after therapy. The control BM samples (*n* = 16) were processed as MRD samples and analyzed using Panels 2 and 3 (Table S2). As for the MRD samples, 500,000 cells were acquired (excluding doublets, debris, and CD45-negative cells). After defining WBCs as the CD45+ population, CD34+/CD117+ and CD34−/CD117+ populations were selected by combining CD45 expression with CD34 and CD117 markers and myeloid markers (CD13, CD33, or HLA-DR), and quantified as a percentage on WBCs. Subsequently, the expression of aberrant antigens was assessed in both studied populations and quantified as a percentage of LAIP+ cells on WBCs (median percentage).

As shown in Table S4 and Figure S4, LAIP rates in both normal and regenerating BM samples were below the MRD interpretative cut-off of 0.035%, with values ranging from 0% to 0.034%. Notably, only the CD123 antigen was expressed at a frequency greater than the threshold. However, since it is not an aberrant lineage antigen, it was not considered for MRD assessments.

### 4.5. Molecular Evaluation of MRD

All 125 BM samples were analyzed for *NPM1* mutations using the RT-qPCR assay based on TaqMan probe chemistry. Total RNA was extracted using the Maxwell^®^ 16 LEV simplyRNA Blood Kit (Promega Corporation, Madison, WI, USA), following the manufacturer’s instructions. Subsequently, RNA was reverse-transcribed into complementary DNA (cDNA) using SuperScript IV Reverse Transcriptase (Life Technologies, Carlsbad, CA, USA). The amplification step was performed using the ipsogen *NPM1* mut A, B&D MutaQuant Kits (QIAGEN, Hilden, Germany), which include pre-designed primers and TaqMan probes specific for the detection of the most common *NPM1* mutations (types A, B, and D). Each RT-qPCR reaction was run in triplicate on an ABI 7900HT Fast Real-Time PCR System (Life Technologies, Carlsbad, CA, USA), with parallel amplification of the ABL1 gene as an internal control. Samples with fewer than 10,000 ABL1 copies were excluded from the analysis to ensure data reliability. Quantification was performed using standard curves provided with the ipsogen *NPM1* MutaQuant Kits, enabling absolute quantification of transcript copy numbers. The *NPM1* expression levels were expressed as the percentage ratio between the mean copy number of *NPM1*-mutated transcripts and the mean copy number of ABL1 transcripts (*NPM1*-mutated ratio). This method is recommended by the European LeukemiaNet (ELN) guidelines for MRD assessment in *NPM1*-mutated AML, as it allows for standardized, reproducible, and clinically meaningful MRD thresholds [2]. Based on previous clinical studies and ELN recommendations [22,23,25,43], a threshold of 0.01% for the *NPM1*-mutated ratio was used to define MRD positivity.

### 4.6. Statistical Analysis

A Chi-squared test was used to evaluate the differences between the concordance of the two MFC methods and *NPM1*-MRD results. Receiving operator characteristic (ROC) curve analysis was performed to evaluate the diagnostic value of the two tests. Concordance, area under the curve (AUC), sensitivity, specificity, and positive/negative predictive values were calculated and compared. Statistical analyses were performed using GraphPad Prism 9 software (GraphPad Software, LLC, San Diego, CA, USA) and R software (version 4.2.2; R Foundation for Statistical Computing, Vienna, Austria).

## 5. Conclusions

Overall, our results demonstrate that the use of the LAIP-based DfN-method improves the accuracy of MFC-MRD analysis in AML patients with the *NPM1* mutation, and emphasize the key role of differentiated evaluation of MRD samples according to therapeutic setting and the definition of different cut-offs to improve MFC-MRD performance. In addition, this MFC-MRD approach is the most appropriate for achieving the best comparability with molecular MRD results.

Prospective clinical studies with a larger sample of AML patients will be needed to correlate these results with the clinical outcome and to translate our specific MFC-MRD approach to other AML patients lacking a molecular MRD target.

## Figures and Tables

**Table 1:** Leukemia-associated immunophenotypes (LAIPs) identified in 25 *NPM1*-mutated AML patients at diagnosis. For each patient, the number of MRD samples (after intensive chemotherapy regimens (post-CHT MRDs, *n* = 62), Ven+HMA-based therapy (post-Ven+HMA MRDs, *n* = 58), and allogeneic stem cell transplant (post-allo-SCT MRDs) and Total MRDs) studied by both MFC and RT-qPCR for *NPM1* mutations, the number of LAIPs identified (only expression of aberrant lineage markers), the percentages of AML cells at diagnosis calculated on white blood cells (WBCs), and the percentage of expression of each biomarker (AML+ cells/total AML cells) were reported. All the LAIPs are highlighted in red, and the aberrant lineage marker most highly expressed by the leukemic cells is highlighted in blue and was selected for the MRD evaluation.

AML Patient	n° Post-CHT MRDs	n° Post-Ven+HMA MRDs	n° Post-Allo-SCT MRDs	n° Total MRDs	n° LAIP	% AML Cells at Diagnosis	CD34	CD117	CD33	CD13	HLA-DR	CD4	CD7	CD56	CD25	CD15	CD123
#1	2	2	0	4	2	47%	10%	100%	100%	100%	100%	**35%**	0%	**58%**	0%	38%	100%
#2	8	0	1	9	2	27%	100%	100%	100%	77%	100%	**85%**	0%	**20%**	0%	0%	0%
#3	2	0	1	3	2	36%	0%	100%	100%	100%	100%	**32%**	0%	**50%**	0%	0%	0%
#4	3	2	1	6	2	30%	5%	100%	100%	85%	100%	**60%**	0%	**24%**	0%	0%	100%
#5	2	5	0	7	3	57%	2%	100%	100%	100%	100%	10%	**64%**	**38%**	**53%**	50%	0%
#6	3	1	0	4	2	14%	50%	100%	100%	100%	100%	**55%**	0%	**25%**	0%	0%	0%
#7	6	0	1	7	2	77%	100%	80%	100%	100%	100%	**35%**	**75%**	0%	0%	0%	0%
#8	3	3	0	6	3	67%	78%	100%	100%	100%	70%	**54%**	**63%**	0%	**67%**	0%	80%
#9	0	5	0	5	2	14%	0%	100%	100%	100%	100%	**68%**	**30%**	0%	0%	0%	0%
#10	7	0	0	7	2	50%	1%	100%	100%	75%	90%	**78%**	2%	**21%**	1%	50%	0%
#11	0	6	0	6	2	44%	0%	100%	100%	78%	100%	**67%**	**22%**	0%	0%	0%	0%
#12	3	1	0	4	2	25%	0%	100%	100%	100%	100%	**32%**	**46%**	0%	0%	0%	0%
#13	8	0	0	8	2	20%	2%	100%	100%	67%	100%	**61%**	0%	**30%**	0%	33%	100%
#14	0	7	0	7	2	60%	10%	100%	100%	60%	100%	**66%**	**22%**	0%	0%	28%	80%
#15	0	2	0	2	2	63%	100%	100%	100%	100%	100%	**50%**	**100%**	0%	0%	0%	0%
#16	0	5	0	5	2	35%	100%	100%	100%	100%	100%	**40%**	**50%**	0%	0%	0%	0%
#17	4	3	0	7	2	20%	0%	100%	100%	42%	100%	**75%**	10%	**20%**	0%	6%	100%
#18	3	3	0	6	3	62%	0%	100%	100%	100%	100%	**32%**	**48%**	**21%**	0%	0%	0%
#19	2	0	0	2	2	82%	100%	100%	60%	85%	100%	11%	**20%**	**50%**	0%	0%	0%
#20	0	4	0	4	2	20%	100%	100%	100%	100%	100%	**20%**	**30%**	0%	0%	0%	0%
#21	2	2	0	4	2	20%	5%	100%	100%	100%	100%	15%	**58%**	0%	**98%**	70%	100%
#22	0	2	0	2	2	21%	8%	100%	100%	100%	100%	**27%**	8%	**37%**	0%	0%	0%
#23	0	2	0	2	2	81%	100%	100%	100%	100%	100%	**32%**	**100%**	0%	0%	0%	0%
#24	0	3	0	3	3	73%	5%	100%	100%	100%	100%	**60%**	**33%**	0%	**95%**	80%	0%
#25	4	0	1	5	2	50%	43%	100%	100%	52%	58%	**48%**	6%	0%	**56%**	25%	0%

## Data Availability

The datasets generated and/or analyzed during the current study are available from the corresponding author on reasonable request (10.5281/zenodo.14983220).

## Supplementary Materials

The following supporting information can be downloaded at: https://www.mdpi.com/article/10.3390/ijms26104506/s1.
