# Supplementary material for: Validation Study of Analytical Methods for Multiparameter Flow Cytometry-Based Measurable Residual Disease Assessment in Acute Myeloid Leukemia
Source: Int J Mol Sci. 2025 May 8;26(10):4506. doi: 10.3390/ijms26104506 (PMC12110934; doi:10.3390/ijms26104506)
Supplement: Supplementary file 1 [file ijms-26-04506-s001.zip › ijms-3584698-supplementary.pdf]

Supplementary Materials

Table S1. Clinical features of AML patients. ELN 2022, European Leukemia Network.

Characteristics	Categories	Values
N° Patients		25
Age (Years), Median (Range)		58 [23-80]
Gender, N° (%)	F	10(40%)
	M	15 (60%)
Diagnosis, N° (%)	De Novo	23 (92)
	Secondary	2 (8)
	Therapy-Related	0 (0)
Eln 2022 Risk Group, N° (%)	Favorable	12 (48)
	Intermediate	11 (44)
	Adverse	2 (8)

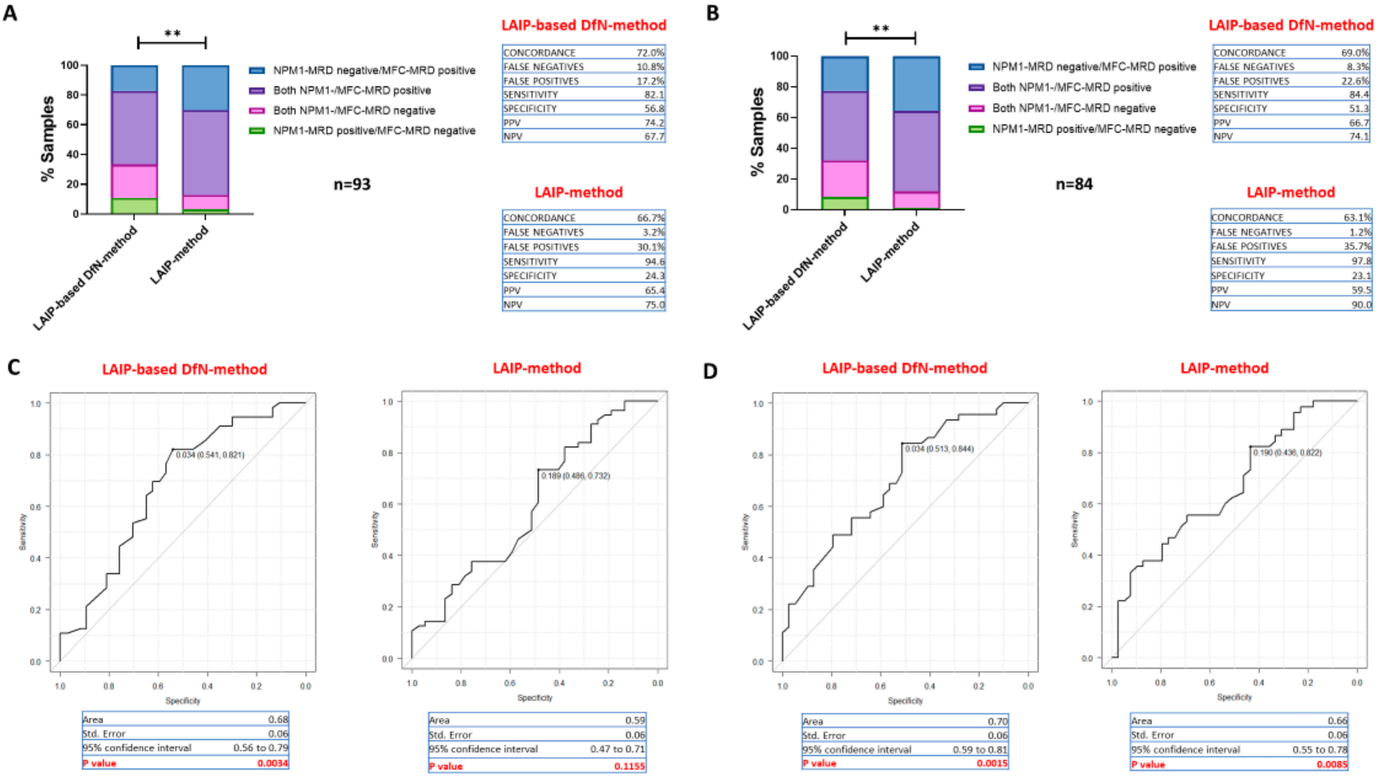

Figure S1. Accuracy of the two MFC-MRD analytical methods in evaluating MRD samples with a total blast percentage (CD34+/CD117+) less than 1% in the MFC analysis and an NPM1 ratio less than 1. A-B: Concordance between the results of MFC-MRD, using LAIP- and LAIP-based DfN-methods, and NPM1-MRD in evaluating MRD samples with a total blast percentage less than 1% for MFC analysis (n=93) and an NPM1 ratio less than 1 (n=84). The results are shown as stacked histogram and expressed as percentage of both positive/negative (purple/pink) and false positive/negative (blue/green) MRD samples (Chi-square test; **p≤0.01). The percentages of concordance, false positive/negative, sensitivity and specificity, and of the positive/negative predictive value (PPV, PNV) of the MFC methods are reported. C-D: ROC curves obtained by distributing the leukemic blast percentages estimated from the LAIP- and LAIP-based DfN-methods according to the NPM1-MRD outcome, analyzing MRD samples with a total blast percentage less than 1% for the MFC analysis (n=93) and with a NPM1 ratio less than 1 (n=84). Area under curve (AUC), Standard Error, 95% confidence interval, and p value of each ROC curve were reported.

A **B**

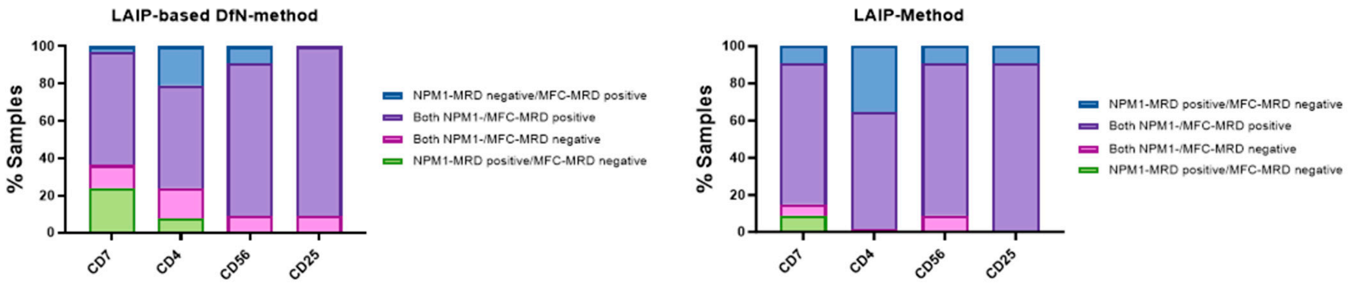

CD7 n=33
CD25 n=11
CD4 n=59
CD56 n=11

Figure S2. Reliability of distinct LAIP-specific aberrant lineage markers using the two MFC-MRD analytical methods in the evaluation of MRD samples from patients with partial LAIP expression (20-90% of AML cells) in AML cells at baseline (n=21 patients; n=114 MRD samples). A-B: Concordance between the results of LAIP-based DfN- and LAIP-methods and NPM1-MRD based on the LAIP-specific aberrant lineage markers used for MRD assessments (CD7 n=33, CD4 n=59, CD56 n=11, CD25 n=11). The results are shown as stacked histogram and expressed as percentage of both positive/negative (purple/pink) and false positive/negative (blue/green) MRD samples.

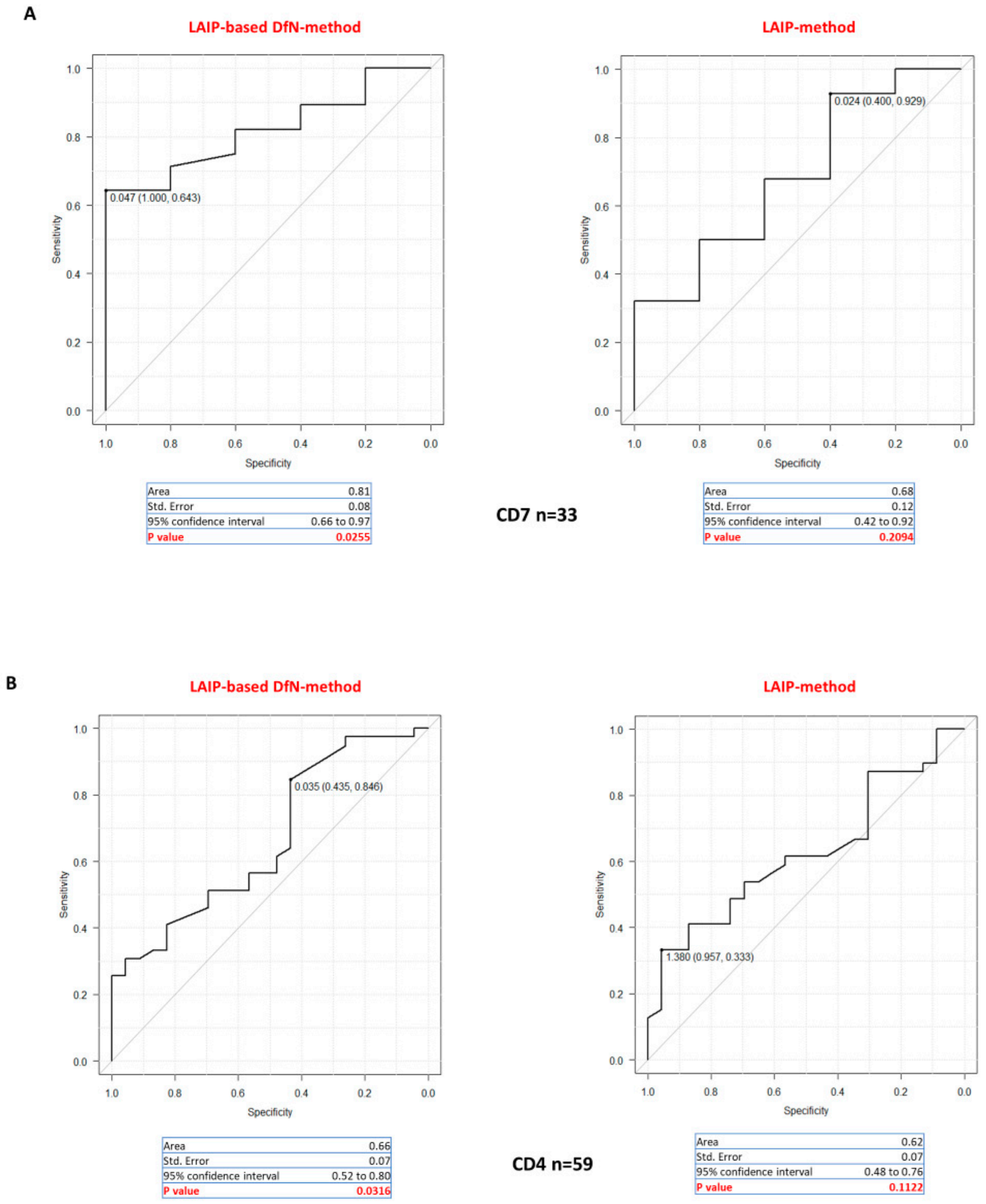

Figure S3. Accuracy of the two MFC-MRD analytical methods based on the LAIP-specific aberrant lineage markers used for the evaluation of MRD samples from patients with partial LAIP expression in AML cells (20-90% of AML cells) at baseline. A-B: ROC curves obtained by distributing the leukemic blast percentages estimated by the LAIP- and LAIP-based DfN-methods according to the NPM1-MRD outcome, analyzing CD7 marker-based MRDs (n=33) and CD4 marker-based MRDs (n=59). Area under curve (AUC), Standard Error, 95% confidence interval, and p value of each ROC curves were reported.

Table S2. Monoclonal antibody (MoAb) panels used for immunophenotyping of AML patients at diagnosis. MoAb clones are reported. Fluorescein isothiocyanate (FITC), Phycoerythrin (PE), Peridinin-chlorophyll-protein-Cyanin5.5 (PerCP Cy5.5), PE- Cyanin7 (PE-Cy7), Allophycocyanin (APC), APC-Hilite7 (APC-H7), Violet 450 (V450), Violet 500 (V500).

Panel	FITC	PE	PerCP Cy5.5	PE-Cy7	APC	APC-H7	V450	V500
1	CD2 (Clone: S5.2)	CD34 (Clone: 8G12)	CD5 (Clone: 17F12)	CD33 (Clone: P67.6)	CD117 (Clone: 104D2)	CD10 (Clone: HI10a)	CD19 (Clone: HIB19)	CD45 (Clone: 2D1)
2	CD33 (Clone: P67.6)	CD13 (Clone: L138)	CD34 (Clone: 8G12)	CD56 (Clone: NCAM16.2)	CD117 (Clone: 104D2)	CD4 (Clone: SK3)	HLA-DR (Clone: L243)	CD45 (Clone: 2D1
3	CD15 (Clone: MMA)	CD123 (Clone: 9F5)	CD34 (Clone: 8G12)	CD25 (Clone: 2A3)	CD117 (Clone: 104D2)	CD7 (Clone: M-T701)	HLA-DR (Clone: L243)	CD45 (Clone: 2D1
4	CD64 (Clone 10.1)	CD4 (Clone: SK3)	CD33 (Clone: P67.6)	CD34 (Clone: 8G12)	CD11B (Clone: D12)	CD14 (Clone: SK3)	HLA-DR (Clone: L243)	CD45 (Clone: 2D1

Table S3. List of internal positive and negative controls used per marker. All populations were gated in the CD45 vs side scatter (SSC-A) plot.

Marker	Positive control	Negative control
CD117	Blasts	Lymphocytes
CD34	Blasts	Lymphocytes
HLA-DR	Monocytes	Granulocytes
CD13	Monocytes/Granulocytes	Lymphocytes
CD33	Monocytes/Granulocytes	Lymphocytes
CD7	Lymphocytes	Monocytes/Granulocytes
CD56	Lymphocytes	Granulocytes
CD4	Lymphocytes/ Monocytes	Granulocytes
CD2	Lymphocytes	Monocytes/Granulocytes
CD25	Lymphocytes	Monocytes/Granulocytes
CD5	Lymphocytes	Monocytes/Granulocytes
CD19	Lymphocytes	Monocytes/Granulocytes
CD10	Lymphocytes	Monocytes
CD15	Monocytes/Granulocytes	Lymphocytes
CD123	Dendritic cells/Monocytes	Lymphocytes
CD14	Monocytes	Lymphocytes
CD11b	Monocytes	Lymphocytes
CD64	Monocytes/Granulocytes	Lymphocytes

Table S4. Median frequencies of LAIPs in normal end regenerating BM samples. The percentage of total CD34+/CD117+ and CD34-/CD117+ cells and of LAIP+ cells for each antigen (CD4, CD56, CD25, CD7, CD15 and CD123) gated on CD34+/CD117+ and CD34-/CD117+ cells in normal (n=7) and regenerating (n=9) BM samples is calculated on CD45+ WBCs and reported as median percentage (minimum-maximun).

	Normal BM samples n=7	Regenerating BM samples n=9
	**Median %**	**Median %**
**CD34+/CD117+**	0,240 (0,017-0,740)	0,200 (0,007-0,632)
**CD34-/CD117+**	0,028 (0,008-0,058)	0,025 (0,005-0,079)
**LAIP**		
**CD34+/CD117+/CD4+**	0,034 (0,002-0,058)	0,032 (0,004-0,119)
**CD34+/CD117+/CD56+**	0,008 (0,001-0,020)	0,007 (0,001-0,090)
**CD34+/CD117+/CD25+**	0,004 (0,000-0,028)	0,022 (0,000-0,076)
**CD34+/CD117+/CD7+**	0,002 (0,000-0,012)	0,008 (0,001-0,022)
**CD34+/CD117+/CD15+**	0,006 (0,000-0,020)	0,009 (0,000-0,065)
**CD34+/CD117+/CD123+**	0,200 (0,013-0,400)	0,150 (0,003-0,462)
**CD34-/CD117+/CD4+**	0,006 (0,001-0,007)	0,003 (0,000-0,014)
**CD34-/CD117+/CD56+**	0,007 (0,002-0,010)	0,002 (0,000-0,015)
**CD34-/CD117+/CD25+**	0,001 (0,000-0,006)	0,000 (0,000-0,007)
**CD34-/CD117+/CD7+**	0,004 (0,001-0,005)	0,002 (0,000-0,007)
**CD34-/CD117+/CD15+**	0,003 (0,000-0,015)	0,004 (0,000-0,033)
**CD34-/CD117+/CD123+**	0,020 (0,003-0,050)	0,013 (0,000-0,027)

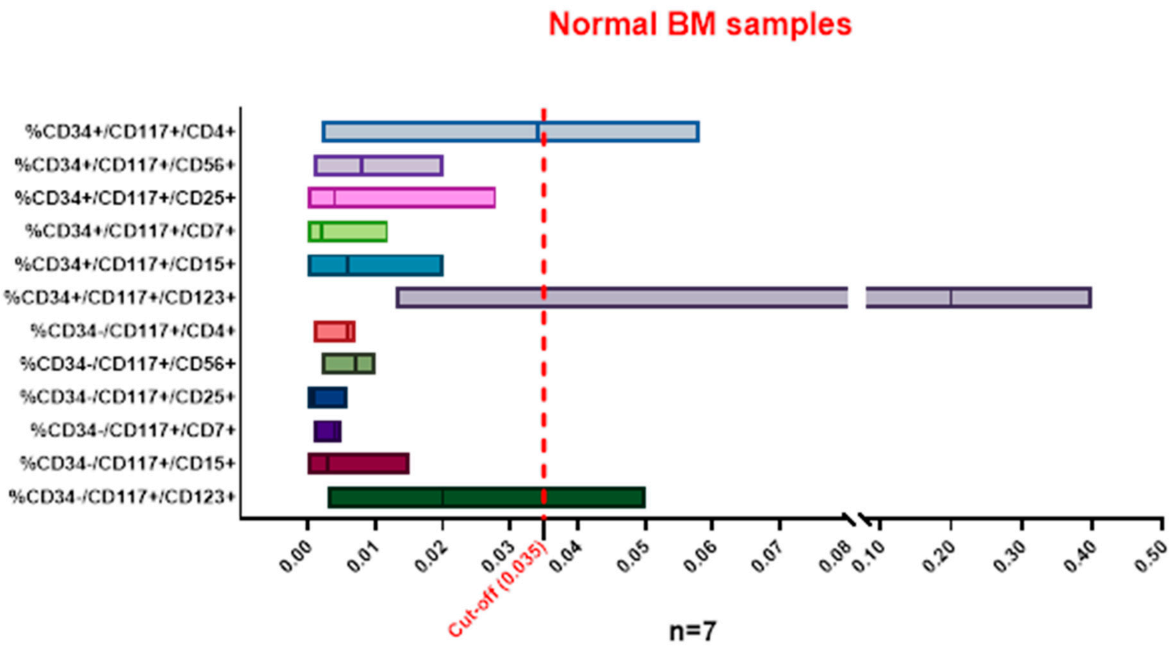

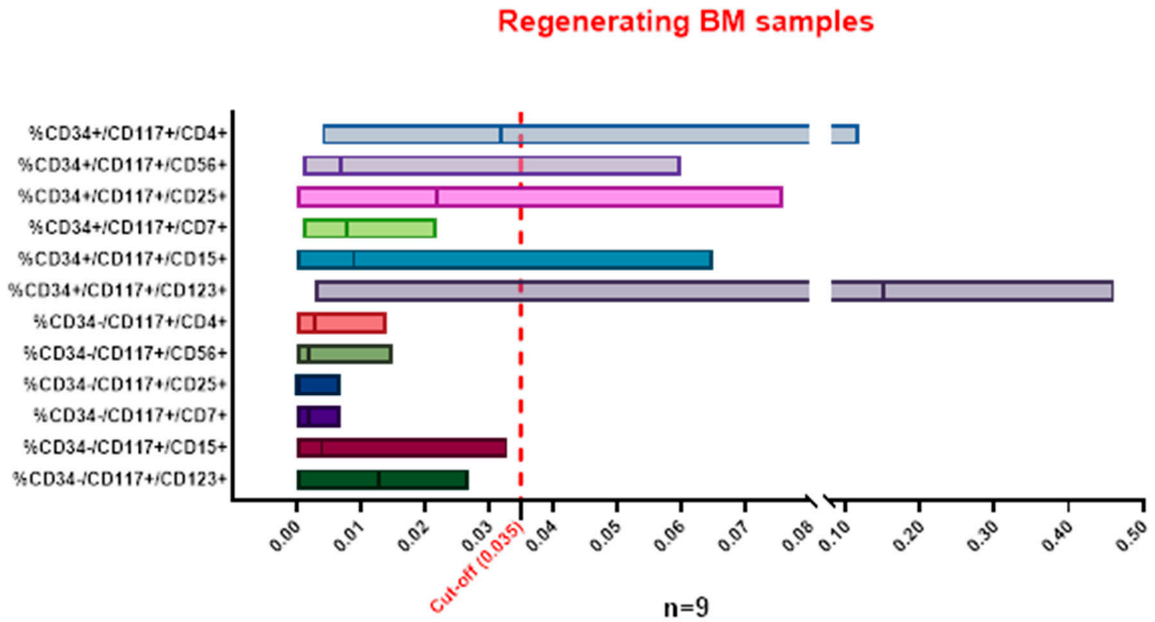

Figure S4. Median frequencies of LAIPs in normal end regenerating BM samples. Representative histogram of LAIP frequencies in normal and regenerating BM samples. Results are represented as floating bars to show the median percentage, minimum and maximum value of each LAIP and expressed as percentage of LAIP+ cells for each antigen (CD4, CD56, CD25, CD7, CD15 and CD123) gated on CD34+/CD117+ and CD34-/CD117+ cells and calculated on CD45+ WBCs. The MRD interpretative cut-off of 0.035% is shown with the red line.
